# Inclined relative magnetic field analysis of Brinkman type dusty fluid through fluctuating upright parallel plates

**DOI:** 10.1016/j.heliyon.2023.e14770

**Published:** 2023-03-28

**Authors:** Dolat khan, Kanayo Kenneth Asogwa, Poom Kumam, Wiboonsak Watthayu, Wiyada Kumam, Musawa Yahya Almusawa

**Affiliations:** a Department of Mathematics, Faculty of Science, King Mongkut's University of Technology Thonburi (KMUTT), 126 Pracha Uthit Rd., Bang Mod, Thung Khru, Bangkok 10140, Thailand; b Department of Mathematics, Nigeria Maritime University, Okerenkoko, Delta State, Nigeria; c Center of Excellence in Theoretical and Computational Science (TaCS-CoE), Faculty of Science, King Mongkut's University of Technology Thonburi (KMUTT), 126 Pracha Uthit Rd., Bang Mod, Thung Khru, Bangkok 10140, Thailand; d Department of Medical Research, China Medical University Hospital, China Medical University, Taichung 40402, Taiwan; e Applied Mathematics for Science and Engineering Research Unit (AMSERU), Program in Applied Statistics, Department of Mathematics and Computer Science, Faculty of Science and Technology, Rajamangala University of Technology Thanyaburi (RMUTT), Pathum Thani 12110, Thailand; f Department of Mathematics, Faculty of Science, Jazan University, Saudi Arabia

**Keywords:** PLPT, Brinkman fluid, Dusty fluid, Inclined relative magnetic field, Fluctuating plate

## Abstract

Due to the widespread use of magnetohydrodynamic (MHD) in electromagnetic targeted therapy, malignant tumor therapy, magnetic microscopy, regulating blood circulation following operations, and fluid pumping in industrial and technical processes. The main goal of the article investigates the analysis of a dusty Brinkman fluid flowing through fluctuating parallel plates with an inclined relative magnetic field. The study aims to analyze the relative magnetic phenomena which is fixed relative to the fluid (MFFRF) or plate (MFFRP), and its impact on fluid and particle motion. The mathematical modelling of the fluid is established through a conventional partial differential equations, and Buckingham's pi theorem is utilized to obtain dimensionless variables. Poincare-Lighthill perturbation technique (PLPT) is employed to derive the solution, with the aid of the program Mathcad-15, the findings are displayed graphically for both velocities. The study indicates that the relative magnetic field significantly influences fluid and particle motion which can be utilized to control fluid pumping in industrial operations and blood flow during surgery.

## 1 Introduction

The fascination with non-Newtonian fluids has risen over the past decade. New studies on non-Newtonian fluids have identified Brinkman-type fluids. Brinkman-type flow is utilized extensively in numerous fields of engineering and science, including aquifer hydrology, geotechnical engineering, rock mechanics, and chemical engineering. Brinkman originally utilized the Brinkman type model in 1949 [1] as a groundbreaking work. Hsu and Cheng [2] investigated fluid flow over a mid-upright plate embedded in a porous structure using the Brinkman model. Ali et al. [3] developed various novel appropriate results for the MHD flow of Brinkman fluid. The remedies were achieved with the help of basic error functions that were complimentary and uncomplicated. Afterwards, Ali et al. [4] analyzed the flow of a Brinkman fluid containing numerous types of spherical nanoparticles and achieved an analytical solution using Laplace transforms. Meanwhile, khan et al. [5] stated the flow of a Brinkman MHD-type viscous fluid in a confined space between two parallel side walls and a plate, considering both transient and steady states. Sheikh et al. [6] investigated the flow of Brinkman-type fluid amongst two plates. They found that as the Brinkman-type fluid parameter values enhanced, the velocity profile decreased. Hasin et al. [7] observed the Brinkman fluid flow across an endless vertical plate. They discovered that adding 4% nanoparticles to clean water increases heat transfer by 17.03%, which will boost thermal performance. In addition, mass transmission falls by 3.18%. Ali et al. [8] examined the flow of Brinkman-type Engine nanofluid in a rotating geometry by employing the Laplace transform approach, thermal radiation and the Hall effect. The addition of MoS2 to engine oil enhanced its lubrication and increased the heat transfer rate by 6.35%. Souayeh et al. [9] studied coupled heat transmission for a hydromagnetic Brinkman fluid undergoing a chemical change in porous media. Imitaz et al. [10] analyzed the dual-phase flow of blood in a cylinder region with an oscillating pressure gradient. They established that, in reaction to an externally provided magnetic field and Brinkman parameter, the velocity profile reduces.

The latest evidence has focused on the two-phase character of dusty liquid simulation dynamics, which has sparked a great deal of curiosity. This phenomenon is observed in fluid (liquid or gas) fluxes including solid particles. The usage of the dusty fluid also be envisioned in rain degradation, precipitation, and missile launchers, among others. These features have accelerated the modelling, solution, and analysis of the migration of dusty fluids. Venkatesh and Kumara [11] studied the uneven transmission of dusty fluids travelling across an undulating wall. They discovered that the dusty particles and fluid are parabolic in composition and continue to approach zero for greater values of time, while the velocities are at their lowest in the channel's middle. Ali et al. [12] examined the oscillating, unstable, viscoelastic fluid of MHD flow with incorporated dust particles. They evaluated the variability of the velocity of the dust and fluid phase. Attia et al. [13] analyzed the power-law highly conductive fluid flow and uninterrupted dusty viscous fluid. They revealed that the particle phase is more affected by the particle viscosity ratio than the fluid phase, as it is the motion of dusty particles. Jalil et al. [14] investigated the dusty fluid flow that conducts electricity across a surface that is extending with an applied magnetic field. By employing the Keller-box method, Arifin et al. [15] analyzed the Jeffrey fluid, the flow behaviour in conjunction with the uniform dispersion of dust particles moving across a vertically contracting sheet. They reported that the momentum and energy curves of dust and fluid phases exhibit a comparable behaviour to all dependent parameters. Bilal et al. [16] tackled the flow of viscoelastic dusty Couette fluid underneath the effect of a transversely generated uniform magnetic field in a rotating system. They claimed that an increase in rotation parameters induces fluid velocity and dust particles to depletion. This is because advancement in the rotation parameter rises the Coriolis force. The researchers Naramgari et al. [17], Nabwey and Mahdy [18] studied the thermal transmission of dusty fluid flow caused by a vertical permeable cone. Higher values of particle phase parameters led to a decrease in momentum curves for fluid temperature, dust-particle and fluid phases, whereas dust-particle phase temperature profiles increased. In addition, both shear and coupling stresses decrease with increasing particle phase parameter mass concentration.

Magnetohydrodynamic (MHD) boundary layer has applications in technology, geological sciences, and engineering linked to numerous mass and heat transport flow concerns that have been investigated. Dar and Elangovan [19] examined the impact of a sinusoidal wave-induced inclination magnetic field on a micropolar fluid in a symmetric channel. They established that raising the inclination angle of the channel boosts the flow rates, whereas the magnetic field enhances the pumping rate. Uwanta et al. [20] swotted the flow of MHD fluid across a vertical surface incorporating Soret and Dufour phenomena. Samiulhaq et al. [21] evaluated the MHD convective transient flow of a 2nd-grade fluid near a vertically saturated porous media. They revealed that magnetic fields dramatically enhance skin friction. Srinivasulu and Goud [22] inspected the outcome of an aligned magnetic field on a deforming surface containing Williamson's nanofluid. They demonstrated that the velocity curves decrease as the slanted magnetic factor augments, but the energy distribution rises as the tilted magnetic parameter rises. Bilal et al. [23] studied the affect of radiant energy on a temperature-constant, inclined electromagnetic surface. Acharya et al. [24] tackled oscillating flows in a porous material using a pointed porous plate. They observed that Lorentz force slows the velocity distribution. Mahato et al. [25] examined an Inclined magnetic impact on nanofluids about a stretching sheet in a porous medium. They found that the nanofluid velocity decreases as the magnetic parameter and tilted angle increase. Shah et al. [26], investigated MHD convective heat transfer with temperature preservation across a moving perpendicular plate in a porous material and found that altering the magnetic field's slope can speed up or slow down fluid flow. On the other hand, Goud et al. [27] examined chemical reactions on MHD flow across a vertical plate using diffusion thermography, while Seth et al. [28] resolved the problem of unsteady hydromagnetic convective flow in a porous medium. Bibi et al. [29] investigated the impact of a dusty tangent hyperbolic fluid numerical study of unstable momentum and heat flow in three dimensions. According to Haider et al. [30], peristaltic wavy movement swotted the MHD solid-liquid two-phase fluid in a revolving tube. By means of the Laplace transform method, Raiz et al. [31] have just published a work on the double-diffusive MHD convective flow. They discovered that, with an MFFRP, fluid velocity does not equal zero at infinity. Zafar et al. [32] studied the MHD free convection flow of a fluid with oscillating motions of an inclined plate and found that the intensity of the magnetic parameter can be utilized to regulate the fluid's motion. To gain a deeper understanding of how MHD can be applied to real-world phenomena, we highly recommend interested readers to refer to Refs. [[33], [34], [35], [36], [37]].

No attempt has been made, to the best of our knowledge, to pinpoint the precise solutions and contrasting effect of fluid and dust particle velocity and temperature distribution for the Brinkman fluid flow with heat transfer along the upright parallel plates, despite a comprehensive review of the aforementioned literature. In light of this, the present work considers the purpose of an inclined relative magnetic field analysis of a Brinkman-type dusty fluid passing through a fluctuating parallel upright plate with wall share stress. MFFRP and MFFRF magnetic phenomena are engaged. Using the PLPT to produce various graphical outputs for velocity and temperature using the MATHCAD-15 package.

## 2 Formulation and solution

**Fig. 1:**
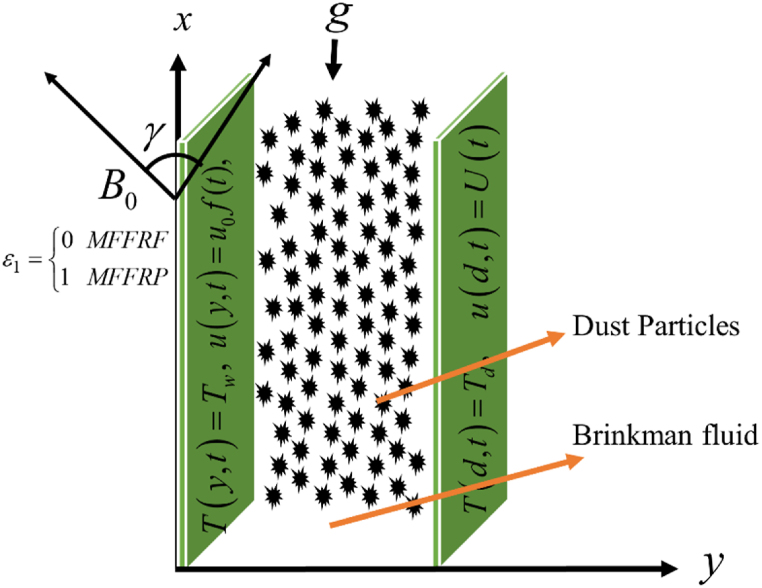
The Problem's physical structure.

Let's consider entirely developed unidirectional, the unsteady flow of electrically conducting viscoelastic, free stream fluctuating Brinkman-type dusty fluid in a vertical channel is explored in this article. The viscoelastic Brinkman-type dusty fluid is flowing with a transverse magnetic field applied. Since of the low magnetic Reynolds number, the electric field resulting from charge polarization and the induced magnetic field are not included i.e. ${Re}_{m}\ll 1$ . Using buoyancy and moving the right plate at free stream velocity U(t) both contribute to the formation of flow. One-dimensional flow beside the x-axis among two adjacent plates has been studied. The right plate oscillates with U(t) while the left plate is rest. The *T*_*p*_ is denotes the particle's temperature as shown in Fig. 1. The outcome of the equations of energy radiation and particle energy are also described. The equation of fluid momentum and mass of a particle, as well as the energy of fluid and particle, can be found by using the assumptions of Bossiness as.

The following are the velocity and temperature fields:

(1) V={u(y,t),0,0},

(2) T={T(y,t),0,0},

The Brinkman fluid constitutional equations are [10,12,34].

(3) $$\nabla \cdot \vec{V}=0\text{,}$$

(4) $$\frac{\partial u}{\partial t}+\beta u=\upsilon \frac{\partial^{2}u}{\partial y^{2}}+\frac{K_{0}N_{0}}{\rho }\left(v-u\right)-\frac{\sigma B_{0}^{2}.\sin\left(\gamma \right)}{\rho }\left(u-\varepsilon_{1}f\left(t\right)\right)+g\beta_{T}\left(T-T_{d}\right)\text{,}$$

(5) $$m\frac{\partial v}{\partial t}=K_{0}\left(u-v\right)\text{,}$$

(6) $$\frac{\partial T}{\partial t}=\frac{k}{\rho c_{p}}\frac{\partial^{2}T}{\partial y^{2}}-\frac{\rho_{p}c_{s}}{\rho c_{p}\gamma_{T}}\left(T_{p}-T\right)-\frac{Q_{0}}{\rho c_{p}}\left(T-T_{d}\right)\text{,}$$

(7) $$\frac{\partial T_{p}}{\partial t}=\frac{1}{\gamma_{T}}\left(T-T_{d}\right)\text{.}$$

The BC’S are;

(8) $$\begin{array}{l} {u\left(y,t\right)|}_{y=0}=u_{0}f\left(t\right),u\left(d,t\right)=U\left(t\right)\\ T\left(0,t\right)=T_{w},T\left(d,t\right)=T_{d} \end{array}\}\text{,}$$

Where $U\left(t\right)=u_{0}\left(1+\frac{\varepsilon }{2}\left(e^{i\omega t}+e^{-i\omega t}\right)\right)$.

We assume the dust particles velocity as:

(9) $$v\left(y,t\right)=v_{0}\left(y\right)e^{i\omega t}\text{,}$$

Using (9) in equation (5), we have

(10) $$v\left(y,t\right)=\left(\frac{K_{0}}{mi\omega +K_{0}}\right)u\left(y,t\right)\text{,}$$

incorporate Eq (10) in Eq (4). So equation (4) becomes,

(11) $$\frac{\partial u}{\partial t}+\beta u=\upsilon \frac{\partial^{2}u}{\partial y^{2}}+\frac{K_{0}N_{0}}{\rho }\left\{\left(\frac{K_{0}}{mi\omega +K_{0}}\right)-1\right\}u-\frac{\sigma B_{0}^{2}\;\sin\left(\gamma \right)}{\rho }\left(u-\varepsilon_{1}f\left(t\right)\right)+g\beta_{T}\left(T-T_{d}\right)\text{,}$$

The subsequent dimensionless variables are incorporated.

(12) $$y^{*}=\frac{y}{d},u^{*}=\frac{u}{u_{0}},t^{*}=\frac{u_{0}t}{d},\theta^{*}=\frac{T-T_{d}}{T_{\omega }-T_{d}},{\theta_{p}}^{*}=\frac{T_{p}-T_{d}}{T_{\omega }-T_{d}},f\left(t^{*}\right)=f_{0}\left(\frac{t^{*}\nu }{u_{0}^{2}}\right)$$

By using Eq. (12), So Eqs (6), (7), (8), (11) become, For the sake of simplicity, the (*) symbol has been disregarded.

(13) $$\frac{\partial u}{\partial t}+\beta u=\frac{\partial^{2}u}{\partial y^{2}}-\left(K_{1}-K_{2}\right)u-M\;\sin\left(\gamma \right)\left(u-\varepsilon_{1}f\left(t\right)\right)+Gr\theta \text{,}$$

(14) $$\frac{\partial \theta }{\partial t}=\frac{1}{Pe}\frac{\partial^{2}\theta }{\partial y^{2}}+\frac{R}{Pe}\left(\theta_{p}-\theta \right)-\frac{\varphi }{Pe}\theta \text{,}$$

(15) $$\frac{\partial \theta_{p}}{\partial t}=\gamma \left(\theta -\theta_{p}\right)\text{.}$$

Having physical dimensionless conditions are:

(16) $$\begin{array}{l} {u\left(y,t\right)|}_{y=0}=f\left(t\right),{u\left(y,t\right)|}_{y=1}=U\left(t\right)\\ \theta \left(0,t\right)=1,{\theta \left(1,t\right)|}_{y=1}=0 \end{array}\}\text{,}$$

Where,

$$M=\frac{\sigma B_{0}^{2}d^{2}}{\rho \upsilon },Gr=\frac{g\beta_{T}\left(T-T_{d}\right)d^{2}}{u_{0}\upsilon },K_{2}=\frac{K_{0}^{2}N_{0}d^{2}}{\rho \upsilon \left(mi\omega +K_{0}\right)},K_{1}=\frac{K_{0}N_{0}d^{2}}{\rho \upsilon },Pe=\frac{u_{0}\rho c_{p}d}{k}\text{,}$$

$$\gamma =\frac{d^{2}}{\upsilon \gamma_{T}},R=\frac{\rho_{p}c_{s}d^{2}}{k\gamma_{T}},\varphi =\frac{Q_{0}d^{2}}{\upsilon \rho c_{p}}\text{.}$$

Where, Gr is Grashof number, $K_{2}$ and $K_{1}$ dusty fluid parameter, β is brinkman parameter, $P_{e}$ Peclet number, φ is a Heat absorption coefficient, M is a magnetic parameter, γ is a time relaxation parameter, R is a parameter for Particle concentration, f(t) is general function.

We assume the solution of Eq (15) as in Eq (17);

(17) $$\theta_{p}\left(y,t\right)=\theta_{p}\left(y\right)e^{i\omega t}$$

(18) $$\theta_{p}\left(y,t\right)=\left(\frac{\gamma }{i\omega +\gamma }\right)\theta \left(y,t\right)\text{,}$$

Put equation (18) in equation (14) get Eq. (19);

(19) $$\frac{\partial \theta }{\partial t}=\frac{1}{Pe}\frac{\partial^{2}\theta }{\partial y^{2}}+\frac{R}{Pe}\left(\left(\frac{\gamma }{i\omega +\gamma }\right)-1\right)\theta -\varphi \theta \text{,}$$

The answer of equation (19) we suppose [14]:

(20) $$\theta \left(y,t\right)=\theta_{0}\left(y\right)+\theta_{1}\left(y\right)e^{i\omega t}\text{,}$$

From equation (20), we get;

(21) $$\theta \left(y,t\right)=\frac{\sin\left(\sqrt{m_{0}}-y\sqrt{m_{0}}\right)}{\sin\left(\sqrt{m_{0}}\right)}\text{,}$$

By incorporating Eq (21) in Eq (13), we get;

(22) $$\frac{\partial u}{\partial t}+\beta u=\frac{\partial^{2}u}{\partial y^{2}}-\left(K1-K2-M\;\sin\left(\gamma \right)\right)u+M\varepsilon_{1}f\left(t\right)+Gr\left\{\frac{\sin\left(\sqrt{m_{0}}-y\sqrt{m_{0}}\right)}{\sin\left(\sqrt{m_{0}}\right)}\right\}\text{,}$$

(23) $$\frac{\partial u}{\partial t}=\frac{\partial^{2}u}{\partial y^{2}}-\left(K1-K2-M\;\sin\left(\gamma \right)-\beta \right)u+M\varepsilon_{1}f\left(t\right)+Gr\left\{\frac{\sin\left(\sqrt{m_{0}}-y\sqrt{m_{0}}\right)}{\sin\left(\sqrt{m_{0}}\right)}\right\}$$

(24) $$\frac{\partial u}{\partial t}=\frac{\partial^{2}u}{\partial y^{2}}-m_{1}u+m_{2}+Gr\left\{\frac{\sin\left(\sqrt{m_{0}}-y\sqrt{m_{0}}\right)}{\sin\left(\sqrt{m_{0}}\right)}\right\}$$

### 3 Momentum equation solution

The solution of equation (22) the Light Hill Technique [14] is used.

(25) $$u\left(y,t\right)=E_{0}\left(y\right)+\frac{\varepsilon }{2}\left(E_{1}\left(y\right)e^{i\omega t}+E_{2}\left(y\right)e^{-i\omega t}\right)$$

(26) $$\begin{array}{l} E_{0}\left(0\right)=f\left(t\right),E_{1}\left(0\right)=0,E_{2}\left(0\right)=0\\ E_{0}\left(0\right)=1,E_{1}\left(0\right)=1,E_{2}\left(0\right)=1 \end{array}\}$$

By incorporating Eq (26) in Eq (25) and using (16) in Eqs. (23), (24), we have $E_{0}\left(y\right),E_{1}\left(y\right)$ and $E_{2}\left(y\right)$ as.

(27) $$\begin{array}{l} E_{1}\left(y\right)=\frac{\sinh\left(y\sqrt{m_{3}}\right)}{\sinh\left(\sqrt{m_{3}}\right)}\text{,}\\ E_{2}\left(y\right)=\frac{\sinh\left(y\sqrt{m_{4}}\right)}{\sinh\left(\sqrt{m_{4}}\right)}\text{,}\\ E_{0}\left(y\right)=H\;\cosh\left(y.\sqrt{m_{1}}\right)+H_{1}\;\sinh\left(y.\sqrt{m_{1}}\right)+n_{0}+n_{1}\left(\frac{\sin\left(\sqrt{m_{0}}-\sqrt{m_{0}}.y\right)}{\sin\left(\sqrt{m_{0}}\right)}\right)\text{,} \end{array}\}$$

Where

(28) $$\begin{array}{l} m_{0}=\frac{Ri\omega +Pe\varphi \left(i\omega +\gamma \right)}{i\omega +\gamma },m_{1}=\left(K_{1}-K_{2}-M\;\sin\left(\gamma_{1}\right)+\beta \right),m_{11}=Pe\left(i\omega -m_{0}\right)\text{,}\\ m_{2}=M\varepsilon_{1}f\left(t\right),m_{3}=m_{1}+i\omega ,m_{3}=m_{1}-i\omega ,n_{0}=\frac{m_{2}}{m_{1}},n_{1}=\frac{Gr}{m_{1}}\text{,}\\ H=f\left(t\right)-n_{0}-n_{1},H_{1}=\frac{1-n_{0}-\left(f\left(t\right)-n_{0}-n_{1}\right)\cosh\left(\sqrt{m_{1}}\right)}{\sinh\left(\sqrt{m_{1}}\right)} \end{array}\}$$

In last we putting the values of $F_{0}\left(y\right),F_{1}\left(y\right),and\;F_{2}\left(y\right)$ in equation (25), we have the form of;

(29) $$u\left(y,t\right)=\left\{\begin{array}{l} H\;\cosh\left(y.\sqrt{m_{1}}\right)+H_{1}\;\sinh\left(y.\sqrt{m_{1}}\right)+n_{0}+n_{1}\left(\frac{\sin\left(\sqrt{m_{0}}-\sqrt{m_{0}}.y\right)}{\sin\left(\sqrt{m_{0}}\right)}\right)+\\ \frac{\varepsilon }{2}\left(\frac{\sinh\left(y\sqrt{m_{3}}\right)}{\sinh\left(\sqrt{m_{3}}\right)}e^{i\omega t}+\frac{\sinh\left(y\sqrt{m_{4}}\right)}{\sinh\left(\sqrt{m_{4}}\right)}e^{-i\omega t}\right) \end{array}\right\}\text{,}$$

Equation (27) satisfies the given physical conditions represents the validity of our computations.

## 4 Results and discussion

**Fig. 2:**
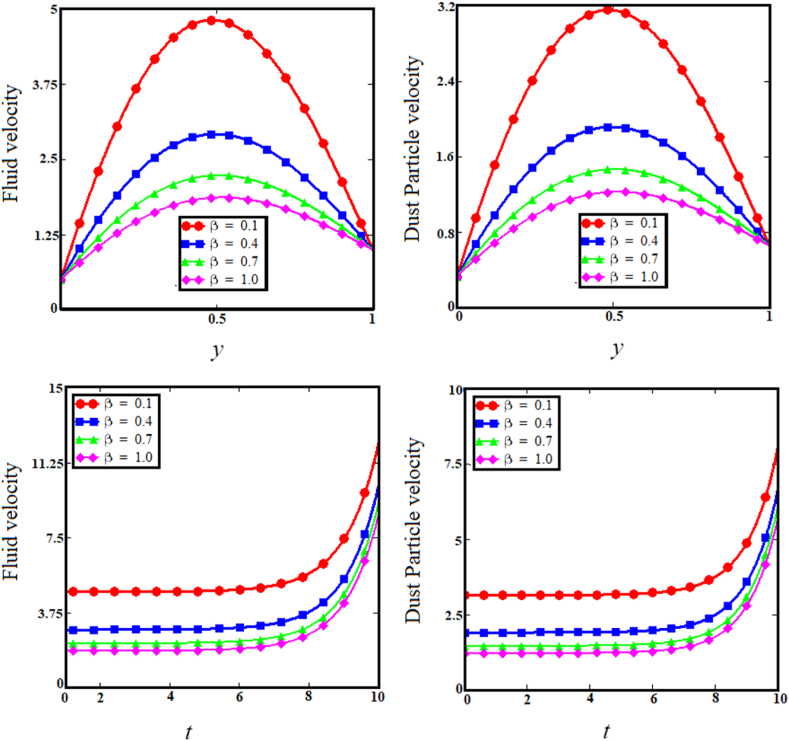
Examining the effects of brinkman parameter on fluid and dust particle profile.

**Fig. 3:**
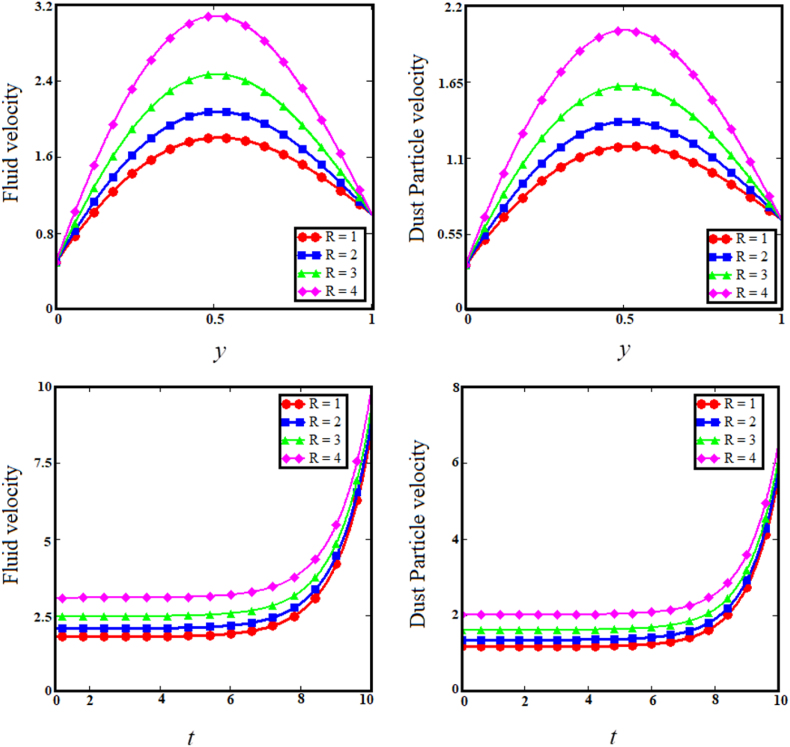
Examining the effects of concentration parameter on fluid and dust particle profile.

**Fig. 4:**
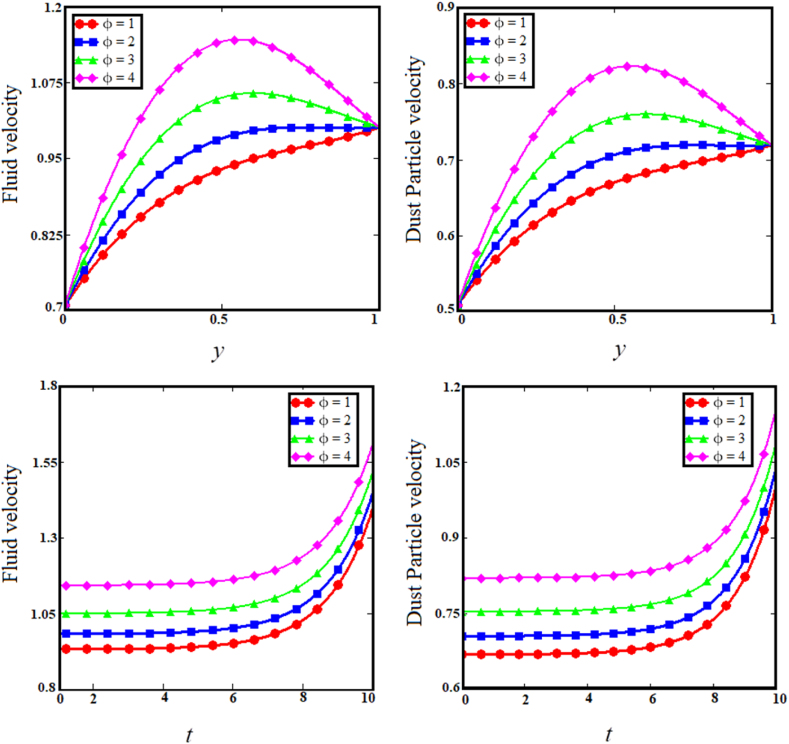
Examining the effects of heat absorption coefficient parameter on fluid and dust particle profile.

**Fig. 5:**
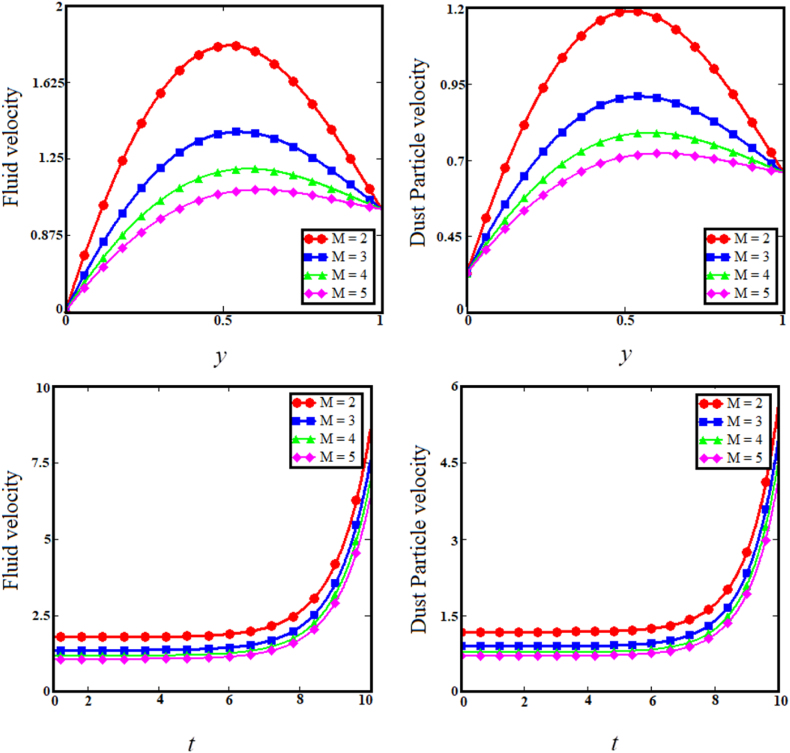
Examining the effects of magnetic parameter on fluid and dust particle profile.

**Fig. 6:**
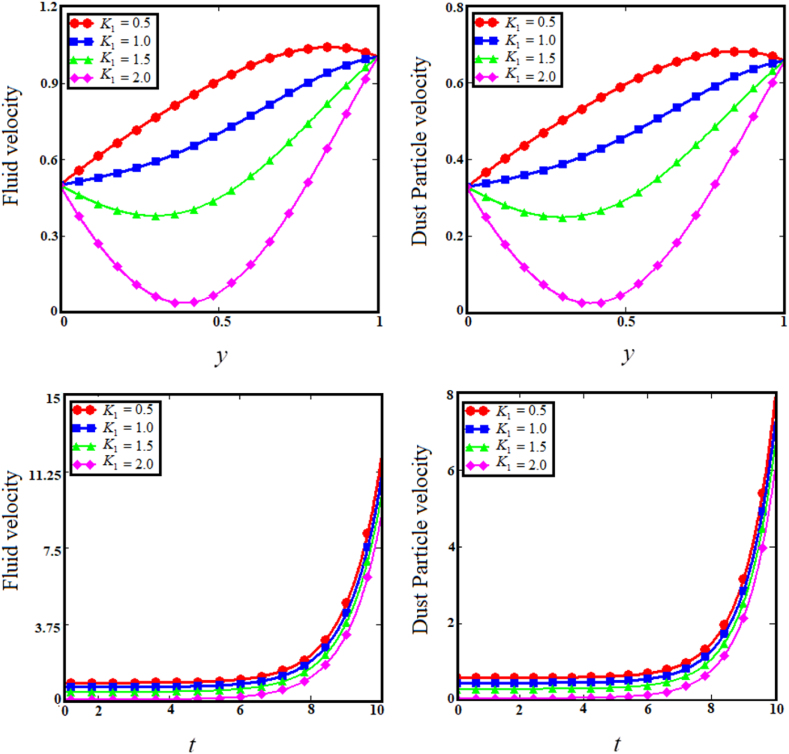
Examining the effects of dusty fluid parameter on fluid and dust particle profile.

**Fig. 7:**
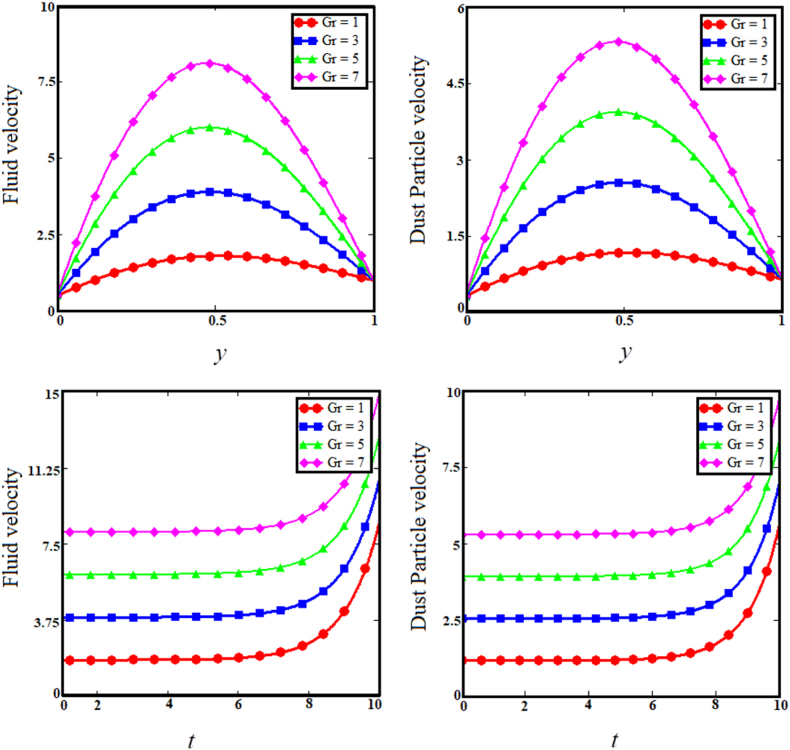
Examining the effects of Grashof number on fluid and dust particle profile.

**Fig. 8:**
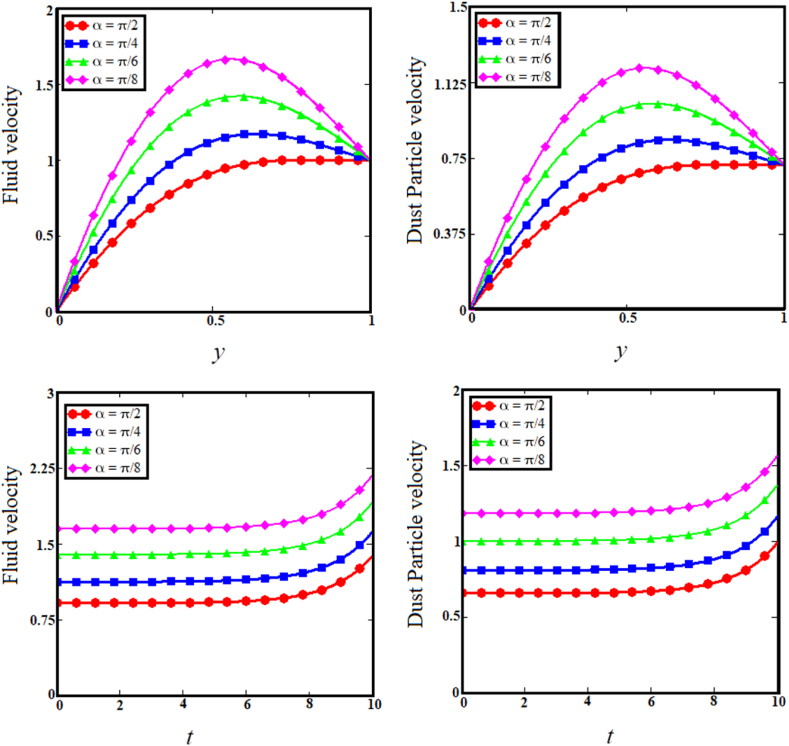
Examining the effects of angle of magnetic field on fluid and dust particle profile.

**Fig. 9:**
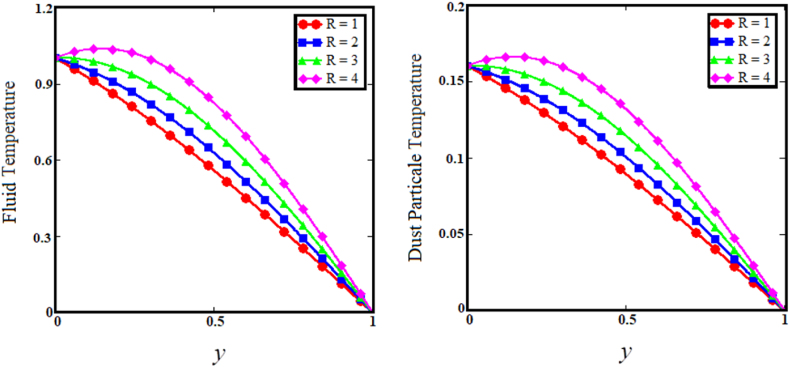
Effect of particle concentration parameter on fluid and dust particle temperature profile.

**Fig. 10:**
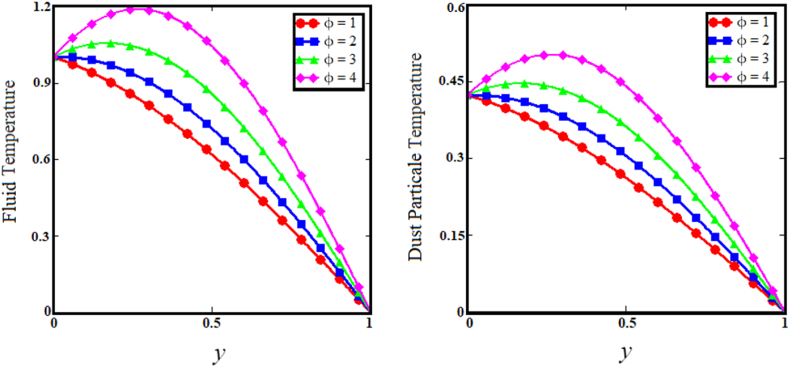
Effect of heat absorption coefficient parameter on fluid and dust particle temperature profile.

**Fig. 11:**
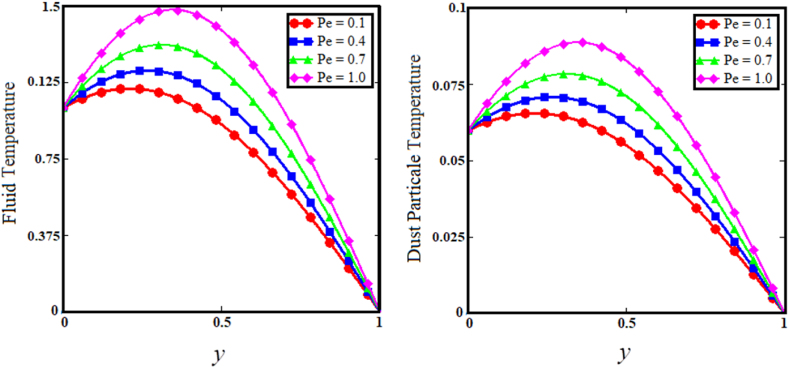
Effect of Peclet number on fluid and dust particle temperature profile.

**Fig. 12:**
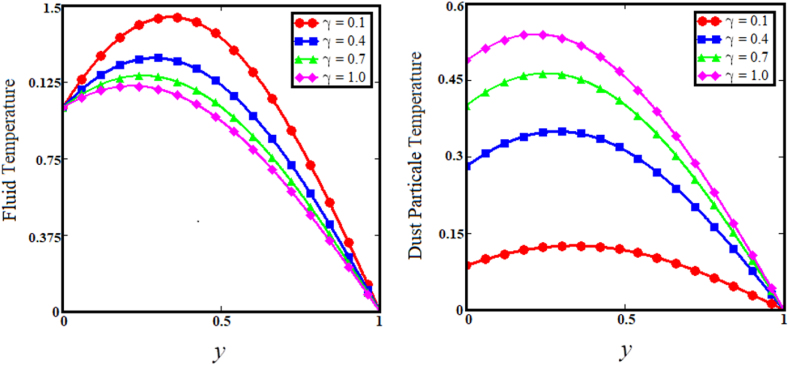
Effect of the temperature relaxation time parameter on fluid and Dust particle temperature.

Keeping in mind the influence of flow parameters on the temperature and velocity profiles, this section is devoted to conveying the physical interpretation of the exact results from inclined relative magnetic field evaluation of Brinkman type dusty fluid using Poincare–Lighthill perturbation technique via a fluctuating vertically parallel plates together with wall share stress, as shown in Fig. 2, Fig. 3, Fig. 4, Fig. 5, Fig. 6, Fig. 7, Fig. 8, Fig. 9, Fig. 10, Fig. 11, Fig. 12.

The two upper sub-graphs of Fig. 2 shows the influence of the Brinkman parameter (β) on the Brinkman fluid velocity and dust particle velocity profiles. As the Brinkman parameter (β) values increase simultaneously for both cases, so does the velocity increase for the fluid and dust particle profiles. Physically, Since the drag forces to density ratio is known as the Brinkman parameter, increasing the drag forces causes the velocity distribution to decrease while the two lower sub-graphs of Fig. 2 shows the velocity versus time for fluid and dust particle with a diminishing influence as the Brinkman parameter increases, validating the boundary conditions for the cases mentioned.

The two upper sub-graphs of Fig. 3 shows the impact of particle concentration parameter (R) on dust particle and fluid velocity distribution. The development in the particle concentration parameter (R) enhances fluid and dust particle velocity distribution. Physically, the particle concentration parameter is inversely proportional to the fluid velocity, because an rise in the concentration of dust particles causes the dusty phase to experience a greater number of collisions if the fluid velocity is greater in proportion, whereas the converse will reduce the fluid's boundary layer. As anticipated, the fluid and dust particle velocities decrease as time passes in the two lower subgraphs of Fig. 3. Since raising the internal restive force decreases the boundary layer velocity with respect to time. Fig. 4 depicts the consequence of the heat absorption coefficient parameter (φ) related to the two upper sub-graphs of fluid and dust particle velocities. The increase in the coefficient of heat absorption parameter (φ) increases dust particle and fluid velocity. Physically, the coefficient of heat absorption is observed at the wall -due to the incremental influence of the heat source on the boundary layer, which significantly increases the fluid and dust particle velocities. The heat observed enhance the kinetic energy of the fluid and dust particles as results the velocity enhance. As the values of the heat absorption coefficient rise, the fluid and dust particle velocities vs time increases, as shown in the lower subgraphs of Fig. 4. The upper sub-graphs of Fig. 5 shows the effect of Magnetic field (M) parameter on fluid velocity and dust particle velocity. It has been noticed that as the Magnetic field parameter enhances, fluid velocity and dust particle velocity decrease. Physically, the magnetic field parameter influences fluid velocity and dust particle velocity, resulting in a decrease caused by the Lorentz force effect. The Lorentz force provides a resistive force that causes fluid and dust particle velocities to encounter resistance. Therefore, decreasing the fluid velocities. In the lower sub-graphs of Fig. 5, a synchronous fall with respect to time is observed for both circumstances. The upper sub-graphs of Fig. 6 displays the impact of dusty parameter $\left(K_{1}\right)$ on the velocity distribution for the dust particle and fluid. The elevation of the dusty parameter dusty parameter $\left(K_{1}\right)$ reduces fluid and dust particle velocity dispersion. As the dusty parameter dusty parameter $\left(K_{1}\right)$ enhances, the dust particle and fluid velocities decrease in the bottom sub-graphs of Fig. 6 along the boundary layer. According to Stocks' drag (k=6πrμ) formula for sphere-shaped dust particles, it is obvious that an increase in the $K_{1}$ would cause the viscous forces to be delayed, which will increase fluid velocity. As a result, boosting the number of dust particles raises and dust particle velocities.

Fig. 7 illustrates the impact of thermal Grashof number (Gr) on the upper sub-graphs of dust particle and fluid velocities. Thermal Grashof number is the viscosity force to buoyancy force ratio. The increase in thermal Grashof (Gr) number enhances the buoyancy force of the fluid velocity and the velocity of dust particles. As the Gr rises, the fluid and dust particle velocities vs time increase rapidly, as shown in the lower subgraphs of Fig. 7. The upper sub-graphs of Fig. 8 illustrate the outcome of the angle of magnetic field parameter on the fluid and dust particle velocity profiles. As the values of the angle of magnetic field parameter increase simultaneously for both situations, so does the velocity for the fluid and dust particle profiles. The lower sub-graphs of Fig. 8 illustrate the velocity versus time for fluid and dust particle with an upward trend as the angle of magnetic field parameter increases. Fig. 9 demonstrates that the rise in the particle concentration parameter (R) enhances fluid and dust particle temperature distribution. Fig. 10 depicts the influence of the heat absorption coefficient parameter (φ) on the fluid and dust particle temperature distribution. The increase in the heat absorption coefficient parameter raises the temperature of the fluid and dust particles. Physically, the coefficient of heat absorption (φ) is observed at the wall due to the incremental influence of the heat source on the boundary layer, which significantly increases both the velocities. Fig. 11 illustrates the relationship between the Peclet number (Pe) and fluid temperature and dust particle temperature. Observations indicate that an increase in Peclet number increases fluid velocity and dust particle velocity. Given the preponderance of viscous forces, the Peclet number (Pe) is the ratio of viscous force to thermal force. Physically, when the Peclet number increases, a greater viscous force on the boundary layer causes an increase in fluid temperature and dust particle temperature. Fig. 12 depicts the influence of the temperature relaxation time parameter (γ) on the fluid temperature and the temperature of dust particles. It is noticed that an rise in the temperature relaxation time parameter decreases fluid velocity while dust particle temperature rises for larger values of the parameter.

**Fig. 13:**
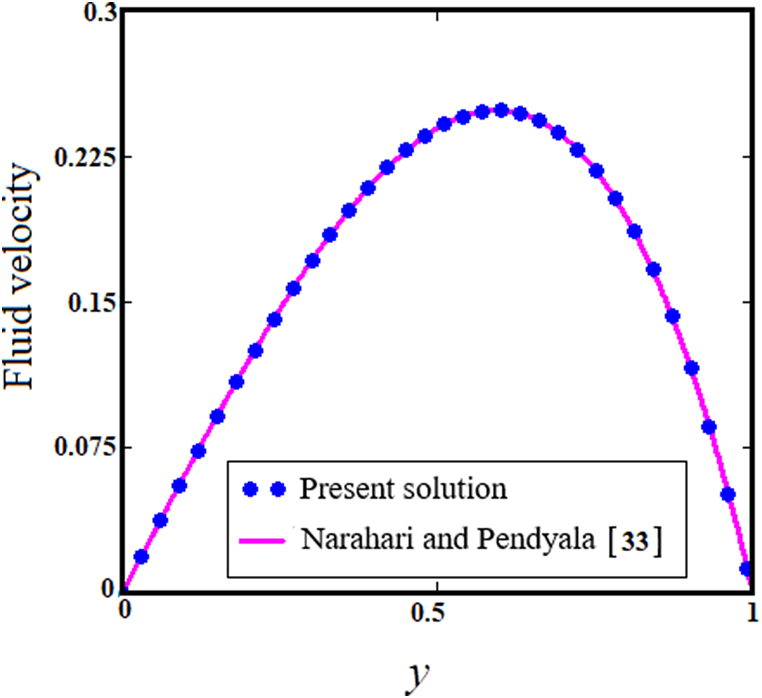
Comparative of existing solutions with Narhari and Pendyala [33].

Fig. 13 shows a comparison of our problem with that of Narahari and Pendyala [33], demonstrating a high degree of consistency. Each solution perfectly overlaps the other, confirming our solutions' soundness and validity.

## 5 Conclusion

The examination of inclined relative magnetic field analysis of Brinkman-type dusty fluid is carried out in a fluctuating upright parallel plate along with wall share stress. Buckingham's pi theorem is utilized in dimensionless equations. The model of the flow is rearranged and then executed via Poincare–Lighthill perturbation technique considering the relative magnetic phenomena of MFFRP or MFFRF to obtain solutions for temperature and velocity profiles in both dust particles and fluid velocity with the support of Mathcad- 15 software. Arising from the study, the key findings listed below are:

• The amplification of the dust particle and fluid velocities is traceable to an upshot of particle concentration parameter (R), coefficient of heat absorption parameter (φ) and thermal Grashof number (Gr).
• Improved values of Brinkman parameter (β), Magnetic field (M), dusty parameter $\left(K_{1}\right)$ on the fluid flow dwindles the dust particle and fluid velocities distribution. The depletion of the fluid and dust particle velocities against time can be noticed when particle concentration parameter (R), heat absorption parameter (φ), Brinkman parameter (β), Magnetic field (M) and dusty parameter $\left(K_{1}\right)$ augment.
• Enhanced values of thermal Grashof number (Gr) and angle of magnetic field parameter (α), accelerate the fluid and dust particle velocities against time.
• The upswing values of particle concentration parameter (R) and Peclet number (Pe) boosts both dust particle and fluid temperature.
• The upsurge of the fluid and dust particle temperature against time can be seen when the heat absorption parameter (φ) grows whereas the contrasting output is noted for the temperature relaxation time parameter.

## Author contributions

Dolat khan, Kanayo Kenneth Asogwa and Wiboonsak Watthayu; conceived and designed the experiments; Dolat khan, Poom Kumam and Wiyada Kumam; performed the experiments; Wiboonsak Watthayu, Wiyada Kumam and Musawa Yahay Almusawa; analyzed and interpreted the data; Dolat khan, Musawa Yahay Almusawa and Poom Kumam; contributed reagents, materials, analysis tools or data; Dolat khan and Kanayo Kenneth Asogwa; wrote the paper.

## Data availability statement

No Data associated in the manuscript.
